# Computational Analysis of nsSNPs of *ADA* Gene in Severe Combined Immunodeficiency Using Molecular Modeling and Dynamics Simulation

**DOI:** 10.1155/2019/5902391

**Published:** 2019-11-03

**Authors:** Soukaina Essadssi, Al Mehdi Krami, Lamiae Elkhattabi, Zouhair Elkarhat, Ghita Amalou, Houria Abdelghaffar, Hassan Rouba, Abdelhamid Barakat

**Affiliations:** ^1^Laboratory of Genomics and Human Genetics, Institut Pasteur Du Maroc, 20360 Casablanca, Morocco; ^2^Laboratory of Biosciences, Integrated and Molecular Functional Exploration (LBEFIM), Faculty of Science and Techniques of Mohammedia, Hassan II University of Casablanca, Casablanca, Morocco

## Abstract

Severe combined immunodeficiency (SCID) is the most severe form of primary immunodeficiency (PID), characterized by fatal opportunistic infections. The *ADA* gene encodes adenosine deaminase, an enzyme that catalyzes the irreversible deamination of adenosine and deoxyadenosine in the catabolic pathway of purine. Mutations of the *ADA* gene have been identified in patients with severe combined immunodeficiency. In this study, we performed a bioinformatics analysis of the human *ADA* gene to identify potentially harmful nonsynonymous SNPs and their effect on protein structure and stability. Using eleven prediction tools, we identified 15 nsSNPs (H15D, H15P, H17Q, H17Y, D19N, T26I, G140E, C153F, A183D, G216R, H258Y, C262Y, S291L, S291W, and K34OE) as harmful. The results of ConSurf's analysis revealed that all these nsSNPs are localised in the highly conserved positions and affect the structure of the native proteins. In addition, our computational analysis showed that the H15D, G140E, G216R, and S291L mutations identified as being associated with severe combined immunodeficiency affect protein structure. Similarly, the results of the analyses of Rmsd, Rmsf, and Rg showed that all these factors influence protein stability, flexibility, and compaction with different levels of impact. This study is the first comprehensive computational analysis of nsSNPs of the *ADA* gene. However, functional analyses are needed to elucidate the biological mechanisms of these polymorphisms in severe combined immunodeficiency.

## 1. Introduction

Severe combined immunodeficiency (SCID) is the most severe form of primary immunodeficiency (PID), a heterogeneous group of hereditary immunological disorders with profound cellular and humoral immunity anomalies, which are characterized by fatal opportunistic infections. This rare disorder occurs in infants and includes life-threatening infections (bacteria, viruses, or fungi), stunting, and diarrhea, and patients usually die within the first two years of life [1].

SCIDs are identified by profound deficiencies in the number and function of T cells, in some types of B and NK cells. Several causes are at the origin of SCID, but genetics is also necessary in the production of this syndrome, which is caused by mutations in one of the genes involved. It has been shown that several genes are involved in severe combined immunodeficiency, such as the *ADA* gene [2].

Severe combined immune deficiency adenosine deaminase (ADA SCID) deficiency represents 10-15% of human SCID cases [1]. The genomic sequence of *ADA* gene spans 32 kb on the long arm of chromosome 20 and contains 12 exons. *ADA* encodes adenosine deaminase, an enzyme that catalyzes the irreversible deamination of adenosine and deoxyadenosine in the purine catabolic pathway [3].

Adenosine deaminase (ADA) deficiency is an autosomal recessive inherited disorder of purine metabolism, which affects lymphocyte development and function. The clinical effects of ADA deficiency are manifest in different organ systems, but most dramatically so in the immune system where it leads to severe lymphopenia with abnormal development of T, B, and natural killer (NK) cells, resulting in reduced cellular immunity and thus a decrease in immunoglobulin production [4, 5].

Bioinformatics is an emerging discipline field mainly involving genetics, molecular biology, computer science, statistics, and mathematics [6].

Single nucleotide polymorphisms (SNPs), one of the main types of genetic variation, are one of the most important resources for understanding the structure and function of the human genome and has become a valuable resource for studying the genetic basis of disease [7].

There were many severe combined immunodeficiency-related genes studied with computational approaches in order to predict functional SNPs such as RAG1, RAG2, and IL2RG to reveal their effect on the structure and function of protein [8, 9].

Currently, more than 70 ADA mutations have been found. Most of them were false sense mutations (63%), 18% were splice mutations, 13% were deletions, and 6% were senseless mutations [10]. These mutations lead to the absence or deficiency of the enzyme adenosine deaminase in the cells, which inhibits the normal degradation of deoxyadenosine. The accumulation of this toxic compound disrupts lymphocyte development and maintenance, which results in severe combined immunodeficiency, a characteristic of adenosine deaminase deficiency [11].

The objective of this study was to perform a computational analysis using a set of mutation prediction tools such as SIFT, PolyPhen-2, and PhD-SNP to identify the most deleterious SNPs and evaluate their pathogenic impact on the protein structure using molecular modelling and molecular dynamics simulation.

## 2. Data and Methods

### 2.1. *ADA* Gene Data Collection

Data of human *ADA* gene was collected from web-based data sources such as Online Mendelian Inheritance in Man (OMIM) and Ensembl (http://asia.ensembl.org/Homo_sapiens/Gene/Summary); the SNP information was derived from the National Center for Biotechnology Information (NCBI) dbSNP (https://www.ncbi.nlm.nih.gov/snp/) and Swiss-Prot (https://expasy.org/) databases.

### 2.2. Analysis and Identification of the Most Damaging SNPs

Many algorithms were used for the functional impact prediction of nonsynonymous single nucleotide polymorphisms (nsSNPs): SIFT [12], PolyPhen-2 [13], PROVEAN [14], M-CAP [15], LRT [16], META SVM, METALR [17], FATHMM-pred, FATHMM-MKL-coding-pred [18], Mutation Assessor [19], and MutationTaster [20].

### 2.3. Evaluation of the Functional Impact of Coding nsSNPs Using a Sequence Homology Tool (SIFT)

SIFT was used to evaluate the functional impact of coding nsSNPs and to predict whether an amino acid substitution in a protein is tolerant or deleterious. SIFT takes a query sequence and uses multiple alignments for the calculation of the probability for all possible substitutions at each position for alignment. Substitutions less than a tolerance index of 0.05 are predicted to be intolerant or deleterious; those greater than or equal to 0.05 are predicted to be tolerated [21, 22].

#### 2.3.1. PolyPhen-2 (Polymorphism Phenotyping)

PolyPhen-2 is a tool for automatically predicting the impact of amino acid substitution on the structure and function of a protein. The program searches for protein 3D structures, using multiple alignments of homologous sequences and amino acid information in several proteins, then calculates position-specific independent count (PSIC) scores for each of two variants, and then computes the difference of the PSIC scores of the two variants. When the difference in the PSIC score is high, the effect of an amino acid substitution is important. PolyPhen-2 predicts as “probably damaging,” “possibly damaging,” or “benign” with the scores 0.95–1, 0.7–0.95, and 0.00–0.31, respectively [23, 24].

#### 2.3.2. Conservation Analysis (ConSurf)

The conservation analysis was realized using the ConSurf web server (http://consurf.tau.ac.il/). It is a tool used to calculate the level of evolutionary conservation of each of the amino acid positions of a protein onto its 3D structure. The conservation scores of each rapidly evolving amino acid position are variable while slowly evolving positions are conserved. The degree of conservation of amino acid is calculated based on a conservational score in the scale of 1-9, where 1-3 contains the most variable positions, 4-6 contains intermediately conserved positions, and 7-9 contains the high conserved positions [25, 26].

#### 2.3.3. Analysis of Structural Impact of SNPs

The FASTA format of the amino acid sequence of ADA was obtained from the UniProt database (https://www.uniprot.org/) [P61764].

The homologous modeling of the native ADA protein was performed by the automated homological modeling on the SWISS-MODEL server. The model used to create the 3D structure was chosen based on the sequence identity and the QMEAN function [27–31]. The 3D mutant structures were produced through the PyMOL software, and the energy minimization for all the 3D structures was done with the GROMACS server [32].

The visualization and analysis of the difference of the hydrogen and hydrophobic bonds between the amino acids of the wild-type protein and its mutated form were done using YASARA software [33].

#### 2.3.4. Molecular Dynamics Simulation

Molecular dynamics (DM) simulations of the structure of the ADA protein and its variants were performed using GROMACS 5.1.4 software with the CHARMM 27 force field [34].

The protein atoms were placed in a cubic box, and other periodic boundary conditions were optimized to perform the simulations. To solvate and neutralize the system, sodium ions were added. Energy minimization was executed using steep descent method for 5000 steps to have a stable conformation.

After minimization, canonical ensembles (NVT) and isobar isothermal ensembles (NPT) were executed with a constant temperature of 300 K for 100 ps for NVT followed by a constant temperature of 300 K and a constant pressure of 1 atm per 100 ps for NPT.

Molecular dynamics simulation was performed at 300 k for 10000 ps. The root-mean-square-deviation (Rmsd), root-mean-square-fluctuation (Rmsf), and radius of gyration (Rg) were calculated by g-rmsd, g-rmsf, and g-Rg [35], respectively. The resulting graphics for these parameters were created using the QtGrace.22 program.

## 3. Results

### 3.1. Distribution of SNPs

**Figure 1:**
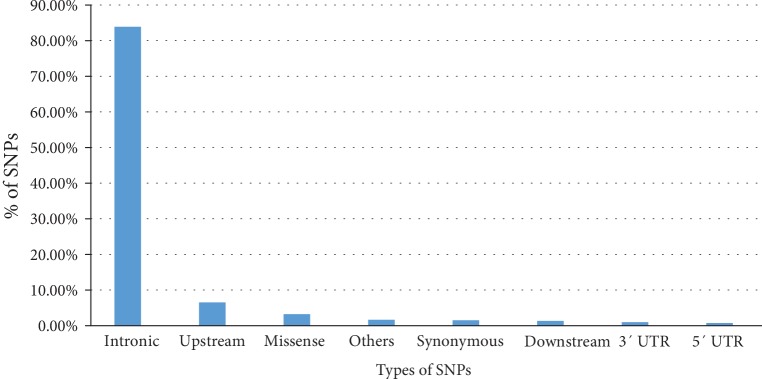
Distribution of SNPs present in the *ADA* gene.

Out of 8557 validated ADA SNPs, 278 SNPs are missense (3.24%), 131 are synonymous (1.5%), 7184 are in the intronic part (83.9%), 63 are in part 5′ UTR (0.73%), 85 are in part 3′ UTR (0.99%), 115 are downstream (1.34%), 559 are upstream (6.53%), and 142 are classified as other types (1.65%) (Figure 1).

### 3.2. The Most Deleterious SNPs Identified in ADA

Several computational tools were used to predict the pathogenic effect of nonsynonymous SNPs on protein structure and function.

Of the 278 nonsynonymous SNPs, only 15 were selected as totally deleterious by the eleven algorithms used: SIFT, PolyPhen-2, PROVEAN, FATHMM, LRT, M-CAP, META SVM, METALR, Mutation Assessor, MutationTaster, and FATHMM-MKL. However, other prediction software have confirmed their deleterious effects. The results are shown in Tables 1 and 2.

### 3.3. Conservation Analysis

Using the ConSurf web server, we analyzed the degree of conservations of ADA residues. The results of the ConSurf analysis showed that 15 deleterious missense SNPs are located in highly conserved regions (7-8-9).

**Figure 2:**
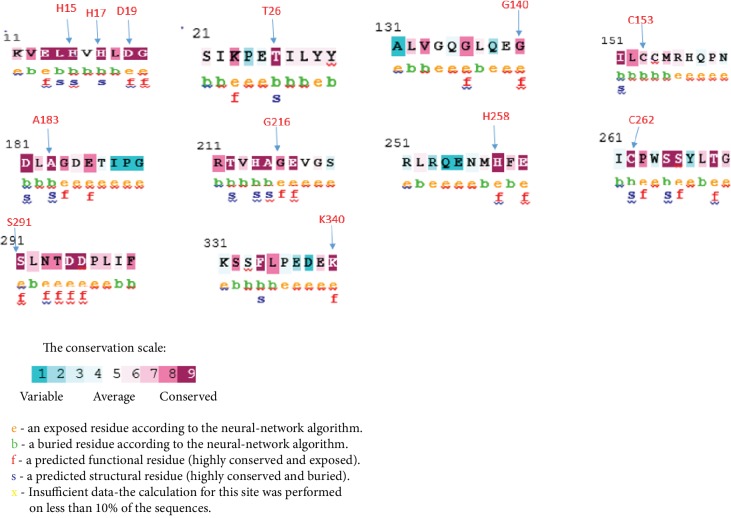
Evolutionary conservation of amino acids in the *ADA* gene determined by the ConSurf server. Value 1 indicates a high variability region. The value increases as the region becomes more conserved, up to value 9.

Among these 15 missenses variants, 14 were located in the highly conserved positions: 7 (D19N, G140E, G216R, H258Y, S291W, S291L, and K340E) were predicted as functional and exposed residues and the other 7 (H15D, H15P, H17Y, H17Q, T26I, A183D, and C262Y) were predicted as buried and structural residues. The C153F was predicted as conserved and buried residue. The results are shown in Figure 2.

### 3.4. Structural Modeling

The SWISS-MODEL server generated two models for ADA (3iar.1.A, 4v7p.1.m). We selected the protein with QMEAN value of 0.10 and 100% identity. The structural analysis of ADA was performed using YASARA software by analyzing the different interactions observed in the mutated and wild-type proteins.

### 3.5. Comparison of Native and Variant Structures of ADA Protein

**Figure 3:**
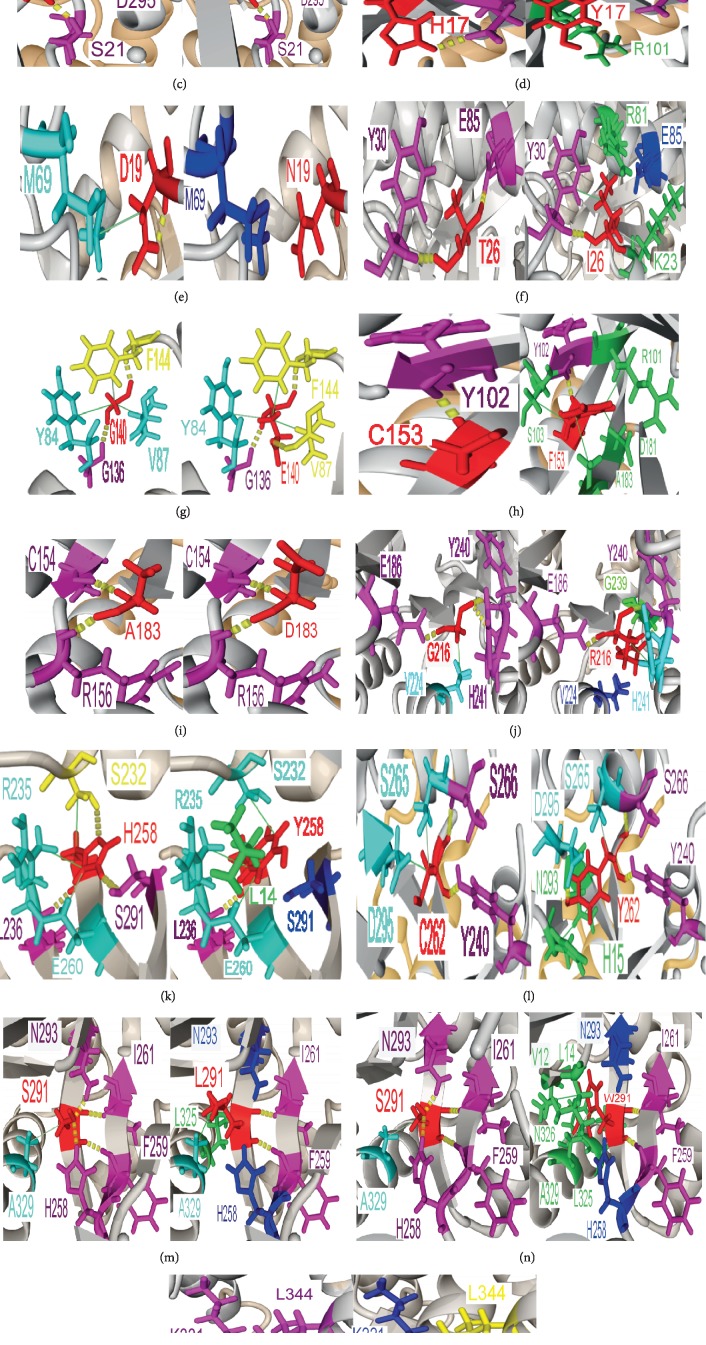
The structural analysis of the different interactions in the mutated protein and the wild-type protein. (a) H15 (wild-type ADA) and 15D (variant protein), (b) H15 (wild-type ADA) and 15P (variant protein), (c) H17 (wild-type ADA) and 17Q (variant protein), (d) H17 (wild-type ADA) and 17Y (variant protein), (e) D19 (wild-type ADA) and 19N (variant protein), (f) T26 (wild-type ADA) and 26I (variant protein), (g) G140 (wild-type ADA) and 140E (variant protein), (h) C153 (wild-type ADA) and 153F (variant protein), (i) A183 (wild-type ADA) and 183D (variant protein), (j) G216 (wild-type ADA) and 216R (variant protein), (k) H258 (wild-type ADA) and 258Y (variant protein), (l) C262 (wild-type ADA) and 262Y (variant protein), (m) S291 (wild-type ADA) and 291L (variant protein), (n) S291 (wild-type ADA) and 291W (variant protein), and (o) K340 (wild-type ADA) and 340E (variant protein). Residues substituted are shown in red; residues involved in hydrogen bonds are marked in magenta; residues that participate in hydrophobic interactions are indicated in cyan; residues that participate in both hydrogen bonds and hydrophobic interactions are marked in yellow; residues which lost hydrogen bonds and/or hydrophobic interactions are marked in blue; and new residues which appeared are indicated in green. Hydrogen bonding is marked by yellow dashed lines, and hydrophobic interactions are shown in green lines.

All the 15 polymorphisms predicted as pathogenic revealed structural changes in the protein by comparing them to the native protein using the YASARA software (Figure 3).

For the variant H15D, we found that the amino acid histidine had two hydrogen bonds with amino acids (D295 and H214), one hydrophobic bond with N293, and two types of hydrogen and hydrophobic interactions with E260. When aspartic acid replaced histidine, the two hydrogen bonds at position 214 and position 260 disappeared (Figure 3(a)).

Regarding the H15P variant, histidine had three hydrogen bonds with amino acids D295, H214, and E260 and two hydrophobic bonds with N293 and E260. When histidine was substituted by proline, the hydrogen bonds with residues D295, H214, and E260 were lost (Figure 3(b)).

Concerning variant H17Q, histidine had two hydrogen bonds with residues D295 and S21; when replaced with glutamine, the hydrophobic bond appeared with residue R101 (Figure 3(c)). Also, for variant H17Y, histidine had two hydrogen bonds with residues D295 and S21; when replaced with tyrosine, two hydrophobic bonds appeared with residues R101 and G20 (Figure 3(d)).

For the D19N variant, aspartic acid had a single hydrophobic bond with methionine at position 69, which disappeared in the mutated protein (Figure 3(e)). For the T26I variant, threonine had two hydrogen bonds with Y30 and F85. When threonine was substituted by isoleucine, three hydrophobic bonds were gained (Y30, R8, and K23), while a hydrogen bond with Y30 was lost (Figure 3(f)). In position 140, glycine had two hydrogen bonds with residues G136 and F144 and three hydrophobic bonds with Y84, V87, and F144. In the mutated protein whose glycine was replaced by glutamic acid, the hydrophobic bond appeared with residue V87 (Figure 3(g)).

In addition, for variant C153F, cysteine established a single hydrogen interaction with residue Y102. When cysteine was replaced by phenylalanine, four hydrophobic bonds were formed (S103, R101, D181, and A183) (Figure 3(h)). In contrast, variant A183D did not reveal any change between the wild-type and mutated proteins (Figure 3(i)).

For variant G216R, glycine had three hydrogen interactions with residues Phe186, Tyr240, and His241 plus one hydrophobic bond with residue Val224. When glycine was substituted by arginine, two hydrophobic bonds were obtained (His241 and Gly239), while two hydrogen interactions with valine and histidine at positions 224 and 241 were, respectively, lost (Figure 3(j)).

About the H258Y variant, the wild-type protein had three hydrogen bonds formed with the three residues S291, L236, and S332 and three hydrophobic bonds with the residues E260, R235, and S332. For the mutated protein, two hydrogen bonds disappeared in positions 291 and 332 (Figure 3(k)).

In addition, for the C262Y variant, cysteine had two hydrogen bonds with amino acids Ser266 and Tyr240 and two hydrophobic interactions with residues Ser265 and Asp295. However, by replacing cysteine with tyrosine, two hydrophobic bonds appeared with H15 and N293 (Figure 3(l)).

The S291L variant had four hydrogen bonds with amino acids N293, I261, F259, and H258 and a hydrophobic bond with the residue Ala329. However, when serine changed to leucine, two hydrogen bonds disappeared with H258 and N293 and a hydrophobic bond appeared with L325 (Figure 3(m)).

It was the same case for wild-type protein of variant S291W, but by replacing serine by tryptophan, two hydrogen bonds disappeared with H258 and N293 and four hydrophobic bonds appeared with L14, V12, N326, and L325 (Figure 3(n)).

For the K340E variant, lysine had four hydrogen interactions with residues L344, K331, E337, and P336 plus a hydrophobic bond with residue L335. When lysine was substituted by glutamic acid, a hydrophobic bond was obtained (L344) and the disappearance of a hydrophobic bond with L335 occurred, while the two hydrophobic interactions with K331 and E337 were lost (Figure 3(o)).

### 3.6. Molecular Dynamics Simulation

The impact of pathogenic SNPs on the protein structure of ADA was assessed by molecular dynamics simulations using GROMACS 5.1.4.

After the generation of the 3D structures of the wild-type protein and its mutated forms, an analysis of the molecular dynamics simulation trajectories for 10000 ps was performed using Rmsd, Rmsf, and Rg.

### 3.7. Stability Analysis

**Figure 4:**
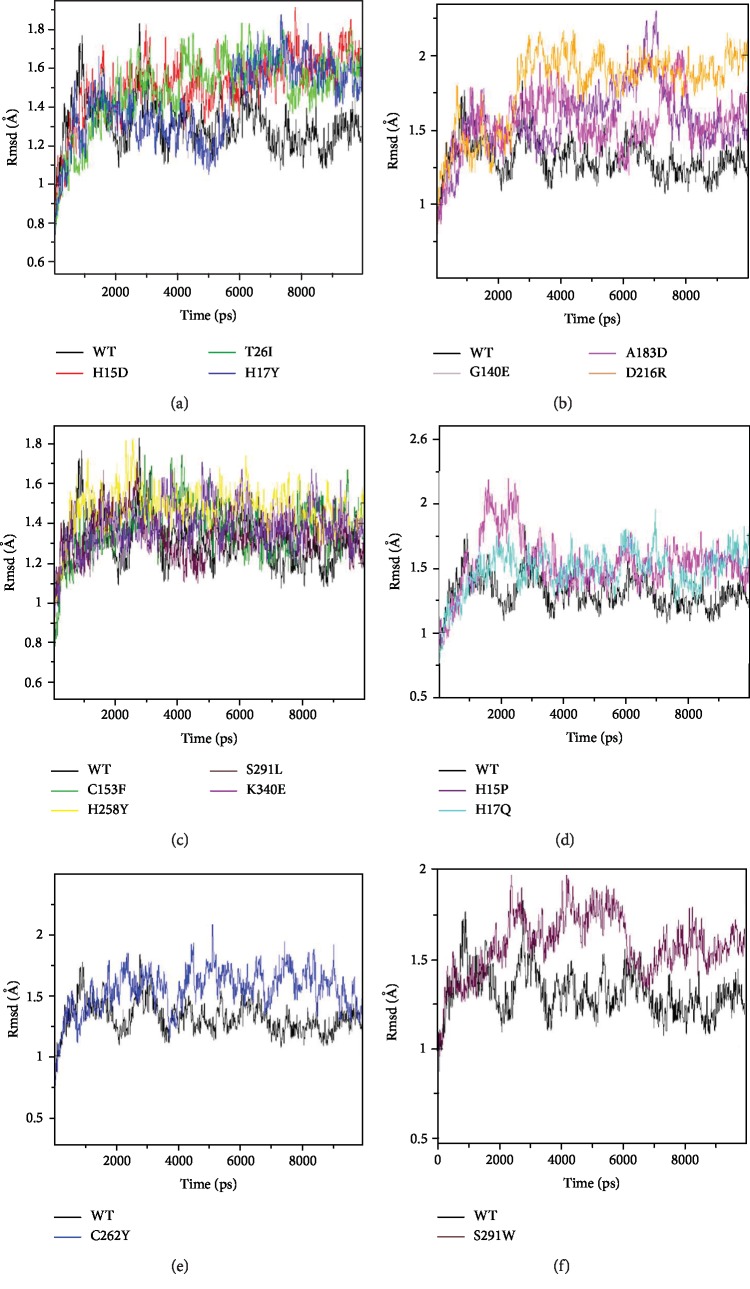
Backbone root-mean-square-deviation (Rmsd) of the wt-ADA protein and variants ADA proteins for 10000 ps molecular dynamics simulation. Color scheme: (a) wt-ADA (black), H15D (red), T26I (green), and H17Y (blue). (b) wt-ADA (black), G140E (violet), A183D (magenta), and G216R (orange). (c) wt-ADA (black), C153F (green), H258Y (yellow), S291L (maroon), and K340E (indigo). (d) wt-ADA (black), H15P (magenta), and H17Q (cyan). (e) wt-ADA (black) and C262 (blue). (f) wt-ADA (black) and S291W (maroon).

At the beginning of the dynamics simulation, the Rmsd value of the native protein ADA was about 1 Å. This value ranged from 1 Å to 1.8 Å during the first to the fourth nanosecond; during the simulation between the fourth and the ninth nanosecond, it ranged from 1 Å to 1.5 Å and decreased during the last nanosecond to 1.2 Å (Figure 4).

For the variants H15D, T26I, H17Y, G140E, A183D, and G216R, their Rmsd values were higher than those of the native protein which varied between 1 Å and 1.8 Å. The H15D variant varied between 1.4 Å and 1.9 Å from 6500 ps to 10000 ps. The H17Y variant diversified between 1.4 Å and 1.8 Å from 6500 ps to 10000 ps, and the A183D variant oscillated between 1.3 Å and 2 Å from 6500 ps to 10000 ps while the native protein diversified between 1 Å and 1.5 Å. The T26I variant varied between 1.3 Å and 1.8 Å from 4000 ps to 10000 ps, and the G140E variant varied between 1.2 Å and 2.2 Å from 4000 ps to 10000 ps, but the native protein varied between 1 Å and 1.5 Å. Then, the G216R variant assorted between 1.5 Å and 2.1 Å from 2500 ps to 10000 ps while the native protein varied between 1 Å and 1.8 Å.

The Rmsd value of the protein with the H15P variant increased to 2.1 Å, from 1000 ps to 2500 ps, while the native protein varied between 1 Å and 1.6 Å. From 6500 ps to 10000 ps, the Rmsd value varied between 1.2 Å and 1.7 Å, and the native form varied between 1 Å and 1.5 Å.

In the H17Q variant, no difference was observed during the first ps up to 6000 ps; at 10000 ps, the trajectory of H17Q showed a significant increase, so the Rmsd value oscillated between 1.1 Å and 1.9 Å, while the Rmsd value of the native protein was between 1 Å and 1.5 Å, during the same period.

For the C262Y variant, the value of Rmsd varied between 1.2 Å and 2 Å, whereas the native protein was between 1 Å and 1.5 Å, during the period 4300 to 9700 ps. The S291W variant varied between 1.4 Å and 1.9 Å from 2000 ps to 60000 ps while the native protein varied between 1 Å and 1.4 Å. From 7000 ps until the end of the simulation, this variant assorted between 1.4 Å and 1.7 Å, but the native protein varied between 1 Å and 1.4 Å.

The C153F, H258Y, S291L, and K340E variants showed a trajectory generally similar to that of the native protein during dynamics simulation.

### 3.8. Analysis of Flexibility

**Figure 5:**
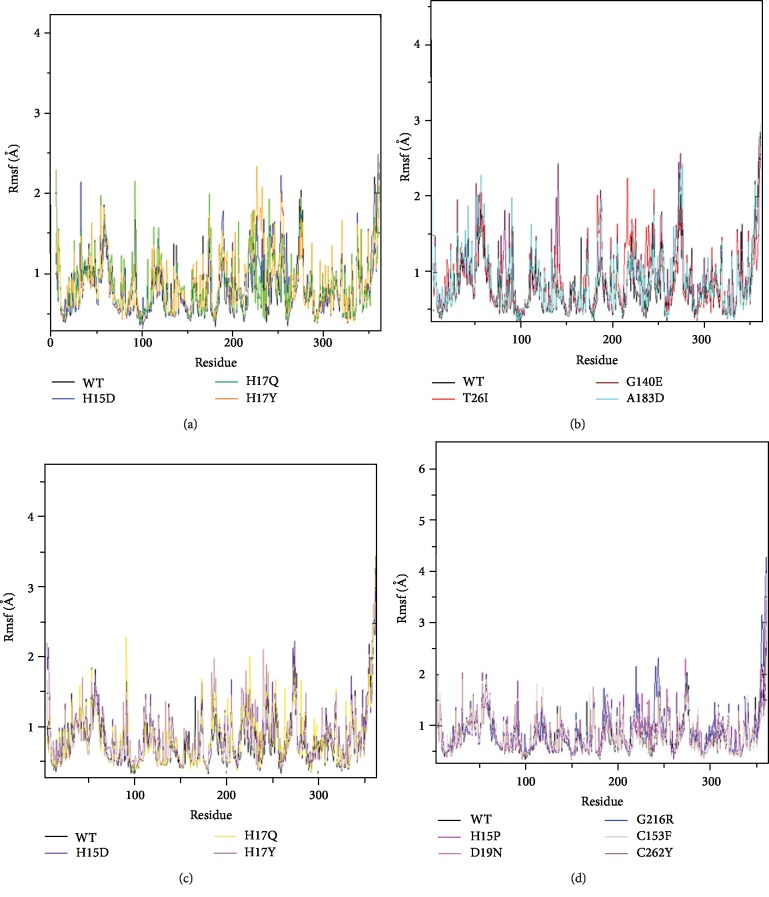
Backbone root-mean-square-fluctuation (Rmsf) of the wt-ADA protein and variants ADA proteins for 10000 ps molecular dynamics simulation. Color scheme: (a) wt-ADA (black), H15D (blue), H17Q (green), and H17Y (orange). (b) wt-ADA (black), T26I (red), G140E (maroon), and A183D (cyan). (c) wt-ADA (black), H258Y (indigo), S291L (yellow), and S291W (brown). (d) wt-ADA (black), H15P (magenta), D19N (violet), G216R (blue), C153F (grey), and C262Y (brown).

The difference in flexibility between amino acids was determined by the analysis of Rmsf during the simulation of molecular dynamics (Figure 5).

The flexibility of the native ADA protein was presented by values between 0.3 Å and 1.8 Å of the amino acid 5 to 100 with 0.3 Å to 1.6 Å between the amino acid 100 to 250 and 0.3 Å to 4 Å from the amino acid 250 to 363.

Concerning the Rmsf values of the *ADA* gene, some variants showed an increase in the Rmsf value compared to wild-type proteins (H15D, H17Q, H17Y, T26I, G140E, A183D, H258Y, and S291L). The H15D variant had Rmsf values between 0.4 and 1.8 amino acid 180 to 190 while the native protein assorted between 0.3 and 1.2, and for the amino acid 270 to 363, the H15D variant had a value of 0.4-3.1, but the wt-ADA varied between 0.3 and 4 in the same period.

In addition, the H17Q variant showed Rmsf values between 0.4 Å and 1.9 Å amino acid 150 to 240 while the native protein showed values between 0.3 Å and 1.5 Å. The H17Y variant oscillated between 0.4 Å and 2.3 Å amino acid 220 to 350 while the native protein of 0.3 Å and 2 Å.

The flexibility of T26I and A183D is presented by values between 0.4 Å and 2.2 Å and 0.3 Å and 2.1 Å of amino acids 170 to 273 while the wt-ADA of values 0.3 Å-1.9 Å. In variant G140E, an Rmsf value of the amino acid 138–275 between 0.3 Å and 2.6 Å was observed, but the native protein was between 0.3 Å and 2 Å.

From the amino acid 200 to 250, the H258Y variant diversified between 0.4 and 1.7, but the native protein between 0.3 and 1.6. For variant S291L, has Rmsf values between 0.3 Å and 2.2 Å amino acid 90 to 230 while the native protein varied between 0.3 Å and 1.6 Å, and variant S291W varied between 0.3 Å and 1.9 Å amino acid 170 to 240 while the native protein was between 0.3 Å and 1.5 Å. No significant difference was reported for variants H15P, D19N, G216R, C153F, and C262Y.

### 3.9. Gyration Analysis

**Figure 6:**
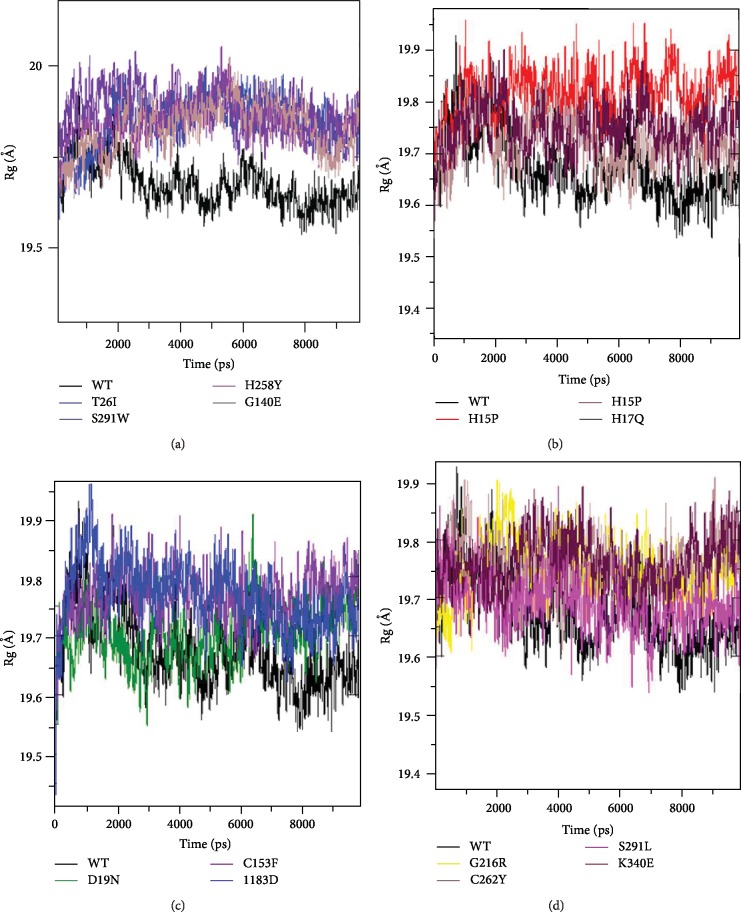
Radius of gyration for wt-ADA and variant proteins over 10000 ps of molecular dynamics simulation. Color scheme: (a) wt-ADA (black), T26I (blue), S291W (violet), H258Y (indigo), and G140E (brown). (b) wt-ADA (black), H15D (red), H15P (brown), and H17Q (maroon). (c) wt-ADA (black), D19N (green), C153 (violet), and A183D (blue). (d) wt-ADA (black), G216R (yellow), C262Y (brown), S291 L (magenta), and K340E (maroon).

The radius of gyration (Rg) analysis was performed to determine the compaction level of each molecule and the overall dimensions of the structure (Figure 6).

At the beginning of the simulation, the Rg value of the native protein (wt-ADA) is about 19.3 Å. During the simulation, the values between 0 and 2500 ps assorted between 19.3 Å and 19.9 Å. After increasing values from 2500 ps up to 10000 ps, values varied between 1.95 Å and 19.7 Å.

The Rg values of the variants T26I, S291W, H258Y, and G140E were significantly higher than those of the native protein. They vary between 19.4 Å and 19.9 Å during the first 2000 ps; after this period until the end of the simulation, 10000 ps diversified between 19.7 Å and 2 Å.

In the middle of simulation, the Rg value of wt-ADA protein was compatible with the Rg value of H15D, H15P, H17Q, H17Y, D19N, C153F, A183D, G216R, C262Y, S291L, and K340E variant proteins.

## 4. Discussion

Computational tools can be used to analyze genetic information and understand genome organization, gene expression, sequence alignment, evolutionary analyses, molecular dynamics, and modeling to study macromolecular structure-to-function relationships [36, 37].

Several studies based on computational tools successfully helped science and medicine. Kumar et al. in 2018 unraveled the impact of missense mutations causing D-2-hydroxyglutaric aciduria 2 using a computational approach that helped them understand the molecular structural change caused by these mutations, and that will serve for the development of novel targets for new drug therapies for this disease [38]. Another study was succeeded using the computational tools as a potential platform, to understand the mechanism of the association between Gaucher's disease and Parkinson's disease, which could facilitate the process of drug discovery against both diseases [39]. In addition, DM simulations could be useful in the development and evaluation of structural models of proteins and protein assemblies [40].

Swiss workers first reported human severe combined immunodeficiency (SCID) more than 50 years ago. Infants with the condition were profoundly lymphopenic and died of infection before their second birthday. The incidence of ADA deficiency is about 1 in 1,000,000 births, but it accounts for 10% to 20% of all cases of SCID. The genomic sequence of *ADA* gene spans to 32 kb on the long arm of chromosome 20 and contains 12 exons. More than 70 ADA mutations have been identified so far [10, 41].

In this study, we conducted an in silico analysis of the human *ADA* gene in order to identify potential deleterious nonsynonymous SNP and their effect on protein structure and stability. SNPs were collected from the dbSNP database. Of the 278 nonsynonymous SNPs, only 15 were selected as totally deleterious by the eleven prediction algorithms used: SIFT, PolyPhen-2, FAHM, Mutation Assessor, MutationTaster, PROVEAN, LRT, M-CAP, FATHMM, META SVM, and METALR.

The set was automatically analyzed by the YASARA visualization software, which visualizes the entire 3D structure of the protein and showed the difference in hydrogen and hydrophobic bonds between the amino acids of the wild-type protein and its mutated forms.

Santisteban et al. examined the genetic basis of adenosine deaminase (ADA) deficiency in seven patients with early or late immune deficiency and identified the substitution of serine at position 291 by amino acid leucine (S291L) in an ADA-deficient SCID patient, who showed improvement during red blood cell transfusion therapy [42].

In addition, another study of the same team identified a new mutation (H15D) found in three unrelated patients with severe combined immune deficiency, the most common phenotype associated with ADA deficiency, and showed that H15D was the first natural mutation of a residue that coordinates directly with the zinc ion associated with the enzyme. Molecular modeling based on the atomic coordinates of the murine ADA suggested that the D15 mutation would create a cavity or space between the zinc ion and the carboxylate of the D15 side chain. This could alter the ability of zinc to activate a water molecule that is supposed to play a role in the catalytic mechanism [43].

Arrendondo-Vega et al. have already identified the substitution of the amino acid glycine by glutamic acid in position 140. This study reported 7 new ADA mutations, with 5 false sense mutations in 7 patients, including 3 with SCID and 4 with late onset. They revealed that the new G140E mutation was probably serious since it occurred in a SCID patient and the degree of elevation of deoxyadenosine nucleotides of AXP > 500 nmol/ml, including the second allele, a previously reported 5 pb suppression, is inactive [44].

Another study by Hirschhorn and colleagues identified a previously unrecognized missense mutation (G216R) in a patient in eastern Pennsylvania with severe combined immune deficiency due to adenosine deaminase deficiency (ADA-SCID) [45].

Molecular dynamics simulation provided detailed information on the stability, flexibility, and overall dimensions of the protein during 10000 ps. The Rmsd's analysis indicated that the H15P, G216R, and C262Y variants showed a high Rmsd value compared to the WT, which decreased the stability of the protein while they had a slight impact on their flexibility and compaction.

The research of Lobanov et al. demonstrated that each class of proteins has its own class-specific radius of gyration, which determines compactness of protein structures; they indicated that alpha-/beta-proteins are the most tightly packed proteins with the least Rg [46]. Our mutants T26I, S291W, H258Y, and G140E revealed an increase in Rg compared to wt-ADA, which means a decrease in their structure compactness which can be caused by the change in their secondary structure.

In addition, protein flexibility increased with variants H15D, H17Q, H17Y, A183D, S291L, and H258Y, but the compaction level of H258Y was lower than WT, while the Rmsd values of the variants H15D, H17Q, and H17Y increased with respect to the WT.

Proteins with the variants T26I, G140E, and S291W have influenced all three parameters: stability, flexibility, and the overall dimensions of proteins.

The analysis of Rmsd, Rmsf, and Rg, following a molecular dynamics simulation, showed that pathogenic SNPs influenced the stability, flexibility, and overall dimensions of proteins. This can disrupt the function of the mutated proteins.

This classification of missense mutations of *ADA* gene could be useful to select the mutants that are worth to be included in experimental studies, to direct gene therapy, and to be targeted by multitargeting drug approaches like strategies based on calixarenes [47, 48] in order to restore the physiological activity of the mutated ADA.

## 5. Conclusion

Computational biology is definitely an effective approach to understand the effect of mutations on protein structure and stability. Several variations have been identified in the *ADA* gene, while the structural and functional impact of many of them has not been analyzed yet to emphasize their involvement in severe combined immunodeficiency.

In this study, 15 nsSNPs were identified as pathogenic variants, which affected the protein structure either by loss or gain of hydrogen and/or hydrophobic interactions, or by influencing parameters such as protein stability, flexibility, and compaction.

Through this study, we demonstrated that these 15 nsSNPs are useful candidates for the detection of mutations associated with SCID within the *ADA* gene. Notably, 4 out of the 15 nsSNPs were found in variant cases according to different in vitro studies as being associated with severe combined immunodeficiency affecting the protein structure.

We hope to provide more information needed to help researchers continue their studies in SCID, especially in our country where consanguinity is common.

## Figures and Tables

**Table 1:** nsSNPs predicted as deleterious by SIFT and PolyPhen.

ID of nsSNPs	AA positions	SIFT	Score	PolyPhen	Score
rs121908725	H15D	Deleterious	0	Probably damaging	1
rs1209280928	H15P	Deleterious	0	Probably damaging	1
rs1270198057	H17Y	Deleterious	0	Probably damaging	1
rs1379847464	H17Q	Deleterious	0	Probably damaging	1
rs1454861940	D19N	Deleterious	0	Probably damaging	1
rs1004808726	T26I	Deleterious	0	Probably damaging	1
rs121908732	G140E	Deleterious	0	Probably damaging	1
rs371028908	C153F	Deleterious	0	Probably damaging	1
rs1163901568	A183D	Deleterious	0	Probably damaging	1
rs121908723	G216R	Deleterious	0	Probably damaging	1
rs1329183956	H258Y	Deleterious	0	Probably damaging	1
rs748088317	C262Y	Deleterious	0	Probably damaging	1
rs121908721	S291W	Deleterious	0	Probably damaging	1
rs121908721	S291L	Deleterious	0	Probably damaging	1
rs769504452	K340E	Deleterious	0	Probably damaging	1

**Table 2:** Confirmation of the deleterious nsSNPs by other prediction software.

AA positions	PROVEAN	FATHMM	LRT	M-CAP	META SVM	METALR	Mutation Assessor	MutationTaster	FATHMM-MKL-coding-pred
H15D	D	D	D	D	D	D	H	D	D
H15P	D	D	D	D	D	D	H	D	D
H17Y	D	D	D	D	D	D	H	D	D
H17Q	D	D	D	D	D	D	H	D	D
D19N	D	D	D	D	D	D	H	D	D
T26I	D	D	D	D	D	D	H	D	D
G140E	D	D	D	D	D	D	H	D	D
C153F	D	D	D	D	D	D	H	D	D
A183D	D	D	D	D	D	D	H	D	D
G216R	D	D	D	D	D	D	H	D	D
H258Y	D	D	D	D	D	D	H	D	D
C262Y	D	D	D	D	D	D	H	D	D
S291W	D	D	D	D	D	D	H	D	D
S291L	D	D	D	D	D	D	H	D	D
K340E	D	D	D	D	D	D	H	D	D

AA: amino acid; D: deleterious; H: high functional.

## Data Availability

All result are included in the article.

## Supplementary Materials

Supplementary Materials Supplementary Table 1: results of the prediction of 278 nsSNPs in human ADA gene using 11 different computational algorithms.
