# Supplementary material for: Computational Analysis of nsSNPs of ADA Gene in Severe Combined Immunodeficiency Using Molecular Modeling and Dynamics Simulation
Source: J Immunol Res. 2019 Nov 3;2019:5902391. doi: 10.1155/2019/5902391 (PMC6875294; doi:10.1155/2019/5902391)
Supplement: Supplementary Materials — Supplementary Table 1: results of the prediction of 278 nsSNPs in human ADA gene using 11 different computational algorithms. [file 5902391.f1.pdf]

Supplementary Materials

Table 1: Results of prediction of 278 nsSNPs in human ADA gene using 11 different computational algorithms.

Id	mutation	SIFT	PolyPhen	FATHMM	LRT	M-CAP	MetaLR	MetaSVM	MutationAssessor	Mutation Taster	PROVEAN	fathmm-MKL_coding
rs1170608999	A359T	T	B	D	N	D	D	T	M	N	N	N
rs749295575	A357G	D	B	D	N	D	D	D	L	N	N	N
rs1313508972	P355T	T	B	D	N	D	D	T	L	N	N	N
rs1398663736	A350S	T	B	D	D	D	D	D	M	D	N	D
rs1327379655	Y348C	T	PD	D	N	D	D	D	M	D	D	D
rs1042207236	Y348H	T	B	D	N	D	D	D	L	D	N	D
rs902376256	L346M	T	B	D	N	D	D	D	L	N, D	N	N
rs925706516	D345E	T	B	D	N	D	D	T	N	N	N	N
rs779530705	D345Y	T	B	D	N	D	D	T	L	N	N	N
rs779530705	D345N	T	B	D	N	D	D	T	N	N	N	N
rs775670821	L343P	D	PD	D	D	D	D	D	H	D	D	D
rs1170562212	L343V	D	PSD	D	D	D	D	D	M	D	N	D
rs1284644841	R341M	D	B	D	N	D	D	T	L	N	N	N
**rs769504452**	**K340E**	**D**	**PD**	**D**	**D**	**D**	**D**	**D**	**H**	**D**	**D**	**D**
rs1379818118	E339G	D	PD	D	D	D	D	D	H	D	D	D
rs867629356	D338N	T	B	D	N	D	D	T	L	N	N	N
rs1262270901	E337G	D	PD	D	D	D	D	D	M	D	D	D
rs1156976510	E337K	D	B	D	N	D	D	D	M	D	D	D
rs1196432166	P336Q	D	PD	D	D	D	D	D	H	D	D	D
rs765787795	P336S	T	PD	D	D	D	D	D	L	D	D	D
rs766550065	S333G	T	PD	D	D	D	D	D	M	D	D	D
rs372131830	K331T	D	B	D	N	D	D	D	M	D	D	D
rs759355046	A330V	D	PD	D	D	D	D	D	M	D	D	D
rs866477587	A330T	D	PD	D	D	D	D	D	H	D	D	D
rs121908715	A329V	D	PD	D	D	D	D	D	H	A	D	D
rs756329749	N328K	D	PD	D	D	D	D	D	M	D	D	N
rs778031507	N328S	D	PD	D	D	D	D	D	M	D	D	D
rs551832248	N328H	D	PD	D	D	D	D	D	M	D	D	D
rs1308151960	I327V	D	PD	D	D	D	D	D	L	D	N	D
rs754097992	R324K	T	B	D	D	D	D	D	L	D	N	D
rs757380249	F322L	T	B	D	D	D	D	T	L	D	N	D
rs765373813	E320K	T	B	D	D	D	D	D	L	D	N	D
rs1208384344	T318S	T	B	D	D	D	D	D	L	D	N	D
rs1319693020	G316S	D	PD	D	D	D	D	D	M	D	D	D
rs889823379	M315I	T	B	D	D	D	D	D	M	D	N	D
rs758501653	M315T	D	PD	D	D	D	D	D	M	D	D	D

rs547569818	D314G	T	B	D	N	D	D	T	N	N, D	N	D
rs923650787	D314Y	T	B	D	N	D	D	T	N	N	N	N
rs747500864	R313Q	T	B	D	N	D	T	T	N	N	N	N
rs79249850	R313W	D	PD	D	N	D	D	D	M	N	D	N
rs748974674	K312R	T	B	D	N	D	D	T	L	N	N	D
rs530131290	K312Q	T	B	D	N	D	D	T	L	N	N	D
rs773914135	T311I	T	B	D	N	D	D	T	N	N	N	D
rs145924854	M310I	T	B	D	N	T	T	T	N	N	N	D
rs1309102513	D305N	T	B	D	N	D	D	T	N	D	N	D
rs199422327	L304R	D	PD	D	D	D	D	D	H	A	D	D
rs1416109540	K301R	T	B	D	D	D	D	T	N	N, D	N	D
rs760574070	F300L	T	B	D	D	D	D	D	M	D	D	D
rs371305751	F300C	D	PD	D	D	D	D	D	M	D	D	D
rs1337332565	F300L	T	B	D	D	D	D	D	M	D	D	D
rs1208759543	I299V	T	B	D	D	D	D	T	N	D	N	D
rs121908718	P297L	D	PD	D	D	D	D	D	H	D	D	D
rs121908718	P297Q	D	PD	D	D	D	D	D	H	A	D	D
rs765445980	D296N	D	PD)	D	D	D	D	D	L	D	D	D
**rs121908721**	**S291L**	**D**	**PD**	**D**	**D**	**D**	**D**	**D**	**H**	**D**	**D**	**D**
**rs121908721**	**S291W**	**D**	**PD**	**D**	**D**	**D**	**D**	**D**	**H**	**D**	**D**	**D**
rs1350879122	Y290C	D	B	D	D	D	D	D	L	D	D	D
rs1017199100	N289T	D	PD	D	D	D	D	D	M	D	D	D
rs1464247052	A288V	T	B	D	N	D	D	T	L	D	N	D
rs752147539	D286E	D	PD	D	D	D	D	D	M	D	D	D
rs781702460	N285Y	D	B	D	N	D	D	D	M	N, D	D	D
rs752935199	L283F	T	B	D	N	D	T	T	N	N, D	N	N
rs751635016	R282L	D	PD	D	D	D	D	D	L	D	D	D
rs751635016	R282Q	T	B	D	D	D	D	D	N	D	N	D
rs542229902	E277D	T	B	D	D	D	D	T	L	N, D	N	D
rs756480576	T276M	D	B	D	N	D	D	D	M	D	N	D
rs1320463424	T276P	D	B	D	N	D	D	D	M	D	D	D
rs778167423	D275E	T	B	D	N	T	D	T	L	N, D	N	N
rs121908738	P274L	T	B	D	D	D	D	D	L	A	D	D
rs1217198230	A271V	D	PD	D	D	D	D	D	M	D	D	D
rs370450947	Y267F	T	B	D	D	D	D	D	M	D	N	D
rs370450947	Y267S	D	PD	D	N	D	D	D	M	D	D	D
rs781040248	S265P	D	PD	D	D	D	D	D	H	D	D	D
**rs748088317**	**C262Y**	**D**	**PD**	**D**	**D**	**D**	**D**	**D**	**H**	**D**	**D**	**D**
**rs1329183956**	**H258Y**	**D**	**PD**	**D**	**D**	**D**	**D**	**D**	**H**	**D**	**D**	**D**
rs1303099065	M257I	T	B	D	D	D	T	T	N	D	N	D
rs145863450	M257T	D	B	D	D	D	D	D	M	D	D	D
rs761098217	E255D	T	B	D	N	D	D	T	L	N	N	N
rs148994526	R253Q	T	B	D	N	D	D	D	M	N	N	N
rs201944717	R253W	D	PD	D	N	D	D	D	M	N, D	D	N
rs762216021	R251S	T	B	D	N	D	D	D	M	D	D	D

rs1254815787	R251W	D	PD	D	N	D	D	D	L	N	D	N
rs765555325	N250D	T	B	D	N	T	D	T	-	N	N	N
rs751244757	Y249C	D	PD	D	D	D	D	D	-	D	D	D
rs754578956	A247T	T	B	D	N	D	D	T	-	N	N	N
rs766952213	D245Y	D	PD	D	D	D	D	D	-	D	D	D
rs766952213	D245N	D	PD	D	D	D	D	D	-	D	D	D
rs1312320956	G239D	D	PD	D	D	D	D	D	-	D	D	D
rs777820729	G239S	D	PD	D	N	D	D	D	-	D	D	D
rs79281338	R235L	D	PD	D	D	D	D	D	-	D	D	D
rs79281338	R235Q	D	PD	D	D	D	D	D	-	D	D	D
rs778809577	R235W	D	PD	D	D	D	D	D	-	D	D	D
rs121908729	T233I	D	B	D	D	D	D	D	-	A	N	D
rs28930973	T233I	D	B	D	D	D	D	D	-	A	N	D
rs772279816	L231F	D	PD	D	D	D	D	D	-	D	D	D
rs775436011	I230V	T	B	D	N	D	D	T	-	N	N	N
rs775436011	I230L	T	B	D	N	D	T	T	-	N	N	N
rs747172905	D229E	T	B	D	D	D	D	T	-	N, D	N	N
rs1347439202	D229G	D	PD	D	D	D	D	D	-	D	D	D
rs768596356	D229H	D	B	D	D	D	D	D	-	D	D	D
rs768596356	D229N	T	B	D	D	D	D	D	-	D	N	D
rs1195013580	V228G	D	PD	D	D	D	D	D	-	D	D	D
rs757210600	V224I	T	B	D	D	D	D	D	N	D	N	D
rs371353841	E222K	T	B	D	N	D	D	T	M	N	N	N
rs747167602	S220L	D	B	D	N	D	D	D	L	D,N	D	D
rs768814204	V218L	T	B	D	N	D	D	D	L	D	N	D
**rs121908723**	**G216R**	**D**	**PD**	**D**	**D**	**D**	**D**	**D**	**H**	**A**	**D**	**D**
rs114025668	A215T	D	PD	D	D	D	D	D	H	D	D	D
rs375707215	T212S	D	PD	D	D	D	D	D	H	D	D	D
rs121908716	R211H	D	PD	D	D	D	D	D	H	A	D	D
rs121908740	R211C	D	PD	D	D	D	D	D	M	A	D	D
rs753338833	H210Y	D	PD	D	D	D	D	D	M	D	D	D
rs761846813	G208S	D	PD	D	D	D	D	D	M	D	D	D
rs750364735	K206N	D	PD	D	N	D	D	D	L	D	N	N
rs758254007	E203D	T	B	D	D	D	D	D	M	D	N	D
rs200089190	E203A	D	B	D	N	D	D	D	L	D	D	D
rs121908734	Q199R	T	B	D	N	D	D	T	N	N, D	N	D
rs121908734	Q199P	D	B	D	N	D	D	T	N	N, D	N	D
rs142456343	H197Q	D	PD	D	D	D	D	D	H	D	D	N
rs762955658	G196E	T	B	D	D	D	D	T	L	D	N	D
rs888003863	G196R	T	PD	D	D	D	D	D	L	D	N	D
rs1272995596	P195R	T	B	D	N	D	D	T	H	N	D,N	D
rs767083081	L193H	T	B	D	N	D	D	T	M	N	N	D
rs1057521456	S192N	T	B	D	D	D	D	D	M	D	N	D
rs1246778030	S192G	T	B	D	D	D	D	D	L	D	N	D
rs767265448	G190E	T	B	D	N	D	D	D	L	N,D	N	N

rs752944142	G184E	D	PD	D	D	D	D	D	H	D	D	D
**rs1163901568**	**A183D**	**D**	**PD**	**D**	**D**	**D**	**D**	**D**	**H**	**D**	**D**	**D**
rs1415459561	A183S	D	PD	D	D	D	D	D	H	D	D	D
rs1421855638	I180T	D	PD	D	D	D	D	D	H	D	D	D
rs121908727	A179D	D	PD	D	D	D	D	D	H	D	D	D
rs779346543	V178A	D	PD	D	D	D	D	D	M	D	D	D
rs746363067	V178L	T	PD	D	D	D	D	D	M	D	D	D
rs121908719	V177M	D	PD	D	D	D	D	D	H	D	D	D
rs770456596	Y172H	D	PSD	D	D	D	D	D	L	D	D	D
rs1042360	K171N	D	PSD	D	D	D	D	D	M	D	D	D
rs371134570	K170E	T	B	D	D	D	D	D	M	D	N	D
rs1203163011	E167D	T	B	D	D	D	D	D	M	D	N	D
rs372671189	E167G	D	PSD	D	D	D	D	D	M	D	D	D
rs1028999099	V165M	T	PSD	D	D	D	D	D	L	D	N	D
rs1414736322	S162C	T	PD	D	D	D	D	D	M	D	D	D
rs1479281280	P159S	T	PD	D	D	D	D	D	M	D	D	D
rs867115027	Q158H	T	B	D	D	D	D	D	L	D	N	D
rs121908722	R156P	D	PD	D	D	D	D	D	H	D	D	D
rs121908722	R156H	D	PSD	D	D	D	D	D	H	A	D	D
rs121908735	R156C	D	PSD	D	D	D	D	D	H	A	D	D
rs28930971	R156C	D	PSD	D	D	D	D	D	H	A	D	D
**rs371028908**	**C153F**	**D**	**PD**	**D**	**D**	**D**	**D**	**D**	**H**	**D**	**D**	**D**
rs121908728	L152V	D	PD	D	D	D	D	D	M	D	D	D
rs121908728	L152M	D	PD	D	D	D	D	D	H	A	N	D
rs28930972	L152V	D	PD	D	D	D	D	D	M	D	D	D
rs28930972	L152M	D	PD	D	D	D	D	D	H	A	N	D
rs990121469	I151T	D	PD	D	D	D	D	D	H	D	D	D
rs121908737	R149Q	D	PD	D	D	D	D	D	M	A	D	D
rs121908733	R149W	D	PD	D	D	D	D	D	H	D	D	D
rs121908733	R149G	D	PD	D	D	D	D	D	L	D	D	D
rs1206634144	A148S	D	PSD	D	N	D	D	D	M	D	N	D
rs551821715	G145E	T	B	D	N	D	D	D	L	N	D	N
rs868364372	D143Y	D	PD	D	D	D	D	D	M	D	D	D
rs61732239	R142P	D)	PSD	D	N	D	D	D	H	D	D	D
rs61732239	R142Q	T	B	D	N	-	D	T	L	D	N	D
rs1199690825	E141K	D	PSD	D	D	D	D	D	M	D	N	D
rs121908732	G140A	D	PD	D	D	D	D	D	M	D	D	D
**rs121908732**	**G140E**	**D**	**PD**	**D**	**D**	**D**	**D**	**D**	**H**	**D**	**D**	**D**
rs746917604	G140R	D	PD	D	D	D	D	D	H	D	D	D
rs1413219982	E139K	T	B	D	D	T	D	D	L	D	N	D
rs1460593121	Q138R	T	B	D	N	T	D	T	N	D	N	D
rs1327639803	G134D	T	PSD	D	N	D	D	T	N	N	N	N
rs121908731	V129L	D	PRD	D	D	D	D	D	H	D	D	D
rs121908731	V129M	D	PD	D	D	D	D	D	H	D	D	D
rs773557479	E128Q	T	B	D	D	D	D	D	M	D	N	D

rs773557479	E128K	D	B	D	D	D	D	D	M	D	D	D
rs7344760	D127E	T	B	D	D	D	D	T	L	D	N	N
rs1210466003	D127N	D	PSD	D	D	D	D	D	M	D	N	D
rs1233957241	P126Q	D	PD	D	D	D	D	D	M	D	D	D
rs1436553912	P126S	D	PD	D	D	D	D	D	M	D	D	D
rs536103858	T125I	D	PD	D	D	D	D	D	H	D	D	D
rs536103858	T125S	T	B	D	D	D	D	D	N	D	D, N	D
rs1163253413	L124I	T	B	D	N	D	D	D	M	D	N	N
rs1458135388	D123H	D	PD	D	D	D	D	D	M	D	D	D
rs748035221	E121V	D	B	D	D	D	D	D	M	D	D	D
rs748035221	E121G	T	B	D	D	D	D	D	M	D	D	D
rs773612521	Q119K	T	B	D	D	D	D	D	-	D	N	D
rs1304346114	N118T	T	B	D	N	D	D	D	-	N	N	D
rs774510141	P116H	D	PD	D	D	D	D	D	-	D	D	D
rs759962237	P116S	T	PSD	D	D	D	D	D	-	D	D	D
rs374166838	P114Q	D	PD	D	D	D	D	D	-	D	D	D
rs1275500780	E113K	T	B	D	N	D	D	T	-	D	N	N
rs998826246	V112A	D	PSD	D	D	D	D	D	-	D	N	D
rs1427855345	V112L	T	B	D	D	D	D	D	-	D	N	D
rs543345924	K111E	T	B	D	D	D	D	T	-	N	N	N
rs1483743360	N109K	D	PD	D	D	D	D	D	-	D	D	D
rs764409246	N109S	T	B	D	N	D	D	D	-	D	D	D
rs1163585601	N109D	D	B	D	D	D	D	D	-	D	D	D
rs121908739	L107P	D	PD	D	D	D	D	D	-	A	D	D
rs267606635	L106V	D	PSD	D	D	D	D	D	-	D	N	D
rs201522960	H105Y	D	PD	D	D	D	D	D	-	D	D	D
rs1452483770	P104L	D	PD	D	D	D	D	D	-	D	D	D
rs1452483770	P104R	D	PD	D	D	D	D	D	-	D	D	D
rs1359688726	Y102H	D	PD	D	D	D	D	D	-	D	D	D
rs28930970	R101L	D	PD	D	D	D	D	D	-	D	D	D
rs28930970	R101P	D	PD	D	D	D	D	D	-	D	D	D
rs28930970	R101Q	D	PD	D	D	D	D	D	-	D	D	D
rs121908717	R101W	D	PD	D	D	D	D	D	-	A	D	D
rs1386689344	V100L	D	B	D	D	D	D	D	-	D	N	D
rs1437613842	E99D	D	PD	D	D	D	D	D	-	D	D	D
rs781241465	V98M	D	PSD	D	D	D	D	D	-	D	N	D
rs267606634	Y97F	D	PD	D	D	D	D	D	-	D	D	D
rs267606634	Y97C	D	PD	D	D	D	D	D	-	D	D	D
rs1316605272	V96M	D	PSD	D	D	D	D	D	M	D	N	D
rs145963969	V95M	D	PD	D	D	D	D	D	H	D	D	D
rs1160387427	E93D	D	B	D	N	D	D	T	L	D	N	D
rs1391023076	A91T	D	PD	D	D	D	D	D	H	D	D	D
rs1344927471	E88A	D	PD	D	D	D	D	D	M	D	D	D
rs778994749	V87A	D	PSD	D	D	D	D	D	M	D	N	D
rs772021681	Y84C	D	PD	D	D	D	D	D	H	D	D	D

rs121908726	A83G	D	PD	D	D	D	D	D	H	D	D	D
rs121908726	A83D	D	PD	D	D	D	D	D	H	D	D	D
rs776103734	A83T	D	PSD	D	D	D	D	D	M	D	D	D
rs1192450607	I82N	D	PD	D	D	D	D	D	H	D	D	D
rs1425739099	R81M	D	PD	D	D	D	D	D	H	D	D	D
rs11555566	K80R	T	B	D	N	-	T	T	N	D	N	D
rs1445758422	A78T	D	PD	D	D	D	D	D	M	D	D	D
rs374983783	R76Q	D	PD	D	D	D	D	D	M	D	D	D
rs121908736	R76W	D	PD	D	D	D	D	D	H	A	D	D
rs747528590	A73V	T	B	D	N	D	D	D	L	D	N	D
rs921477673	A73T	D	B	D	D	D	D	D	M	D	N	D
rs148785262	I72F	D	PSD	D	D	D	D	D	M	D	D	D
rs148785262	I72V	T	B	D	N	D	D	D	L	D	N	D
rs762213530	A71V	T	B	D	N	D	T	T	N	N	N	N
rs968895664	P70S	T	PSD	D	D	D	D	D	L	D	D	D
rs752086551	F61L	T	PD	D	D	D	D	D	M	D	D	D
rs760262095	D60G	T	B	D	N	D	D	T	N	N	N	N
rs760262095	D60A	T	B	D	N	D	D	T	N	N	N	N
rs77588173	P55L	D	B	D	D	D	D	D	M	D	D	D
rs750682305	K54R	T	B	D	N	D	D	D	L	N	N	D
rs1294381082	D53N	T	B	D	N	D	D	D	L	N	N	D
rs934757654	M52K	T	B	D	D	D	D	D	L	D	N	D
rs1057460440	I50T	D	PD	D	D	D	D	D	M	D	D	D
rs1306348962	V49I	T	B	D	N	D	D	T	N	N	N	N
rs1276626543	N48S	T	B	D	N	D	D	T	N	N	N	N
rs1363563144	G45E	T	B	D	N	D	D	T	N	N	N	N
rs780318972	T42R	D	PSD	D	D	D	D	D	M	D	D	D
rs199961890	L38F	T	PD	D	D	D	D	D	H	D	D	D
rs151336936	A37V	T	B	D	N	D	D	T	L	N	N	N
rs1044335093	A37T	T	B	D	N	D	D	T	N	N	N	N
rs748611869	I36M	D	B	D	D	D	D	D	M	D	N	D
rs770213537	I36T	D	PSD	D	D	D	D	D	M	D	D	D
rs1431003280	I36L	T	B	D	D	D	D	D	L	D	N	D
rs376909062	G35R	D	PSD	D	D	D	D	D	M	D	D	D
rs1251027847	R34T	D	PD	D	D	D	D	D	M	D	D	D
rs773388108	R33K	T	B	D	N	D	D	T	N	D	N	N
rs1356820014	R32K	T	B	D	N	D	T	T	N	N	N	N
rs759080719	Y30C	D	PD	D	N	D	D	D	L	N	N	D
rs775324175	Y30H	T	PD	D	N	D	D	D	L	N	N	D
rs746622099	Y29C	D	PSD	D	D	D	D	D	M	D	D	D
rs199886437	L28V	T	B	D	D	D	D	D	L	D	N	D
rs1455312539	I27T	D	PD	D	D	D	D	D	H	D	D	D
**rs1004808726**	**T26I**	**D**	**PD**	**D**	**D**	**D**	**D**	**D**	**H**	**D**	**D**	**D**
rs761815086	P24T	T	B	D	D	D	D	D	M	D	N	D
rs1321058509	I22F	D	B	D	D	D	D	D	M	D	D	D

rs765144557	S21C	D	B	D	D	D	D	D	L	D	N	D
rs139350872	S21A	T	B	D	N	D	D	T	N	N	N	N
rs121908724	G20R	D	PD	D	D	D	D	D	H	D	D	D
rs762695968	D19E	D	PD	D	D	D	D	D	M	D	D	D
**rs1454861940**	**D19N**	**D**	**PD**	**D**	**D**	**D**	**D**	**D**	**H**	**D**	**D**	**D**
**rs1379847464**	**H17Q**	**D**	**PD**	**D**	**D**	**D**	**D**	**D**	**H**	**D**	**D**	**D**
**rs1270198057**	**H17Y**	**D**	**PD**	**D**	**D**	**D**	**D**	**D**	**H**	**D**	**D**	**D**
rs1330122216	V16A	D	B	D	D	D	D	D	M	D	D	D
rs1330122216	V16D	D	PSD	D	D	D	D	D	L	D	D	D
**rs1209280928**	**H15P**	**D**	**PD**	**D**	**D**	**D**	**D**	**D**	**H**	**D**	**D**	**D**
**rs121908725**	**H15D**	**D**	**PD**	**D**	**D**	**D**	**D**	**D**	**H**	**D**	**D**	**D**
rs73598374	D8Y	D	PSD	D	N	D	D	D	L	D	N	D
rs73598374	D8H	D	PSD	D	N	D	D	D	L	D	N	D
rs73598374	D8N	T	B	D	N	-	T	T	N	N	N	N
rs956908942	A6S	T	B	D	N	D	D	D	N	D	N	D
rs956908942	A6P	T	B	D	N	D	D	D	N	D	N	D
rs1464312281	P5S	D	B	D	N	D	D	D	N	D	N	D

D: Deleterious; H: High functional; PD: Probably damaging; PSD: Possibly damaging;

T: Tolerated; B: Benign; N: Neutral; M: Medium; L: Low
